# The use of on-site visits to assess compliance and implementation of quality management at hospital level

**DOI:** 10.1093/intqhc/mzu026

**Published:** 2014-03-25

**Authors:** C. Wagner, O. Groene, M. Dersarkissian, C.A. Thompson, N.S. Klazinga, O.A. Arah, R. Suñol, on behalf of the DUQuE Project Consortium

**Affiliations:** 1 NIVEL Netherlands Institute for Health Services Research, Utrecht, The Netherlands; 2 Department of Public and Occupational Health, EMGO Institute for Health and Care Research, VU University Medical Center, Amsterdam, The Netherlands; 3 Department of Health Services Research and Policy, London School of Hygiene & Tropical Medicine, London, UK; 4 Department of Epidemiology, Fielding School of Public Health, University of California, Los Angeles (UCLA), Los Angeles, CA, USA; 5 Palo Alto Medical Foundation Research Institute, Palo Alto, CA, USA; 6 Department of Public Health, Academic Medical Center, University of Amsterdam, Amsterdam, The Netherlands; 7 UCLA Center for Health Policy Research, Los Angeles, CA, USA; 8 Avedis Donabedian Research Institute (FAD), Universitat Autonoma de Barcelona, Barcelona, Spain; 9 Red de Investigación en Servicios de Salud en Enfermedades Crónicas (REDISSEC), Barcelona, Spain

**Keywords:** quality management, audit, implementation, on-site visits, hospital

## Abstract

**Objective:**

Stakeholders of hospitals often lack standardized tools to assess compliance with quality management strategies and the implementation of clinical quality activities in hospitals. Such assessment tools, if easy to use, could be helpful to hospitals, health-care purchasers and health-care inspectorates. The aim of our study was to determine the psychometric properties of two newly developed tools for measuring compliance with process-oriented quality management strategies and the extent of implementation of clinical quality strategies at the hospital level.

**Design:**

We developed and tested two measurement instruments that could be used during on-site visits by trained external surveyors to calculate a Quality Management Compliance Index (QMCI) and a Clinical Quality Implementation Index (CQII). We used psychometric methods and the cross-sectional data to explore the factor structure, reliability and validity of each of these instruments.

**Setting and Participants:**

The sample consisted of 74 acute care hospitals selected at random from each of 7 European countries.

**Main Outcome Measures:**

The psychometric properties of the two indices (QMCI and CQII).

**Results:**

Overall, the indices demonstrated favourable psychometric performance based on factor analysis, item correlations, internal consistency and hypothesis testing. Cronbach's alpha was acceptable for the scales of the QMCI (*α*: 0.74–0.78) and the CQII (*α*: 0.82–0.93). Inter-scale correlations revealed that the scales were positively correlated, but distinct. All scales added sufficient new information to each main index to be retained.

**Conclusion:**

This study has produced two reliable instruments that can be used during on-site visits to assess compliance with quality management strategies and implementation of quality management activities by hospitals in Europe and perhaps other jurisdictions.

## Introduction

In a more and more market-oriented health-care delivery system it may be increasingly important to evaluate the quality of care delivered. To this end, health-care purchasers are gathering and using performance information on patient experiences as well as organizational and clinical performance indicators. Despite years of efforts to improve the reliability and validity of performance indicators, there are still differences in outcomes related to registration and measurement error in the administrative databases used to calculate outcome indicators. Additional information about quality management and clinical quality strategies could be a valuable adjunct to hospital assessment, especially since accreditation/certification instruments that can be used to assess quality management systems already exist [1].

An alternative to administrative data and surveys is the on-site visit (or audit). Visits involving independent auditors can verify compliance with activities, methods and procedures used to plan, control, monitor and improve the quality of care. On-site visits are mainly used by accreditation and certification organizations who visit an organization for a few days, reviewing documents and meeting with front-line staff. To date, easy-to-use survey instruments have not been developed for use by health-care purchasers or health-care inspectorates. Such instruments could be used to reveal whether a hospital has appropriate quality management strategies in place, whether they are used and whether they are stimulating continuous learning and improvement. The latter is based on the Deming or Nolan Quality Improvement Cycle, which describes the steps: Plan–Do–Check/study–Act. On-site visits offer an opportunity to discuss achievement of more complex steps like ‘Check and Act’. In addition, widely used quality indicators can rarely be used to measure the improvement structures and culture of hospital units, which can be explored in conversation during an on-site-visit.

In this article we describe the development of two novel quality management indices for purchasers and other stakeholders of European hospitals. Both are developed within the DUQuE project (Deepening our understanding of Quality Improvement in Europe). One (the Quality Management Compliance Index or QMCI) focuses on compliance with existing quality management procedures at the hospital level and the other (the Clinical Quality Implementation Index or CQII) on activities that support continuous improvement of clinical indicators. This paper describes testing of the psychometric properties of the two newly developed measurement instruments (QMCI and CQII).

## Methods

### Setting and participants

The study took place in the context of the DUQuE project which ran from 2009 to 2013 [2]. Hospitals were sampled at random from all acute care hospitals in seven European countries: Czech Republic, France, Germany, Poland, Portugal, Spain and Turkey. Data for the QMCI and CQII were collected in the hospitals that participated in the in-depth study of the DUQuE project. In total, 74 hospitals (response rate 88%) were visited by experienced surveyors and these responses were used for the psychometric analyses.

### Development of the instruments

#### Quality Management Compliance Index

The aim of the QMCI was to identify and verify the compliance to a set of closely related methods and procedures used to plan, monitor and improve the quality of care. By design, three scales of the index were defined *a priori* as quality planning, monitoring opinions of professionals and patients, and improvement of quality of care based on the notion that if a hospital would like to base its quality management on a limited number of activities, then it would need to have a plan based on the opinions of front-line staff and the users of hospital services, patients. Furthermore, strategies are necessary to solve possible shortcomings mentioned by professionals and patients.

The choice of questionnaire items was based on expert opinion of experts with years of experience in hospital performance evaluation during accreditation and certification audits. The main criteria for including an item were its assumed influence on quality and safety of care, and the feasibility of verifying an answer to the item. Face validity was established based on the review by 10 experts of the DUQuE project, and a pilot test in two hospitals. All items of the QMCI (*n* = 15) were rated on a five-point Likert scale, varying from ‘no or negligible compliance’ (0) to ‘full compliance’ (4).

#### Clinical Quality Implementation Index

The purpose of the CQII is to test clinical quality systems and seek evidence of their implementation at the hospital level. The CQII has been designed to measure to what extent efforts regarding key clinical quality areas are implemented across the hospital. Following Bate and Mendel [3], each quality effort is assessed with regard to three levels of development: (i) Do quality efforts regarding the key areas exist (i.e. is there a responsible group and hospital protocol)? (ii) To what extent are these efforts monitored (i.e. with regard to compliance and improvement measurements)? (iii) To what extent is the sustainability of these efforts monitored?

The key clinical areas included stem from the different quality functions described in most accreditation systems, as well as the recommendations of the WHO Patient Safety Alliance covering most of the key hospital clinical and safety areas. In total, seven areas were selected: (i) preventing hospital infection, (ii) medication management, (iii) preventing patient falls, (iv) preventing pressure ulcers, (v) routine assessment and diagnostic testing of patients in elective surgery, (vi) safe surgery that includes an approved checklist and (vii) preventing deterioration and advance life support (i.e. rapid response teams, resuscitation programmes). As a whole, the audit instrument comprised items from seven clinical areas, which could be rated on a five-point Likert scale, varying for example from ‘no compliance’ to ‘full compliance’. Additionally, when no information was available ‘not applicable’ could be selected. However, to get more meaning groups, the answer categories were recoded to a scale of 1–3, where responses of no, negligible or low compliance were coded as 1, medium compliance was coded as 2 and high, extensive or full compliance were coded as 3. The choice for the seven clinical areas was based on the fact that evidence exists on how to prevent the unsafe practices and related adverse outcomes. By following the existing guidelines patient harm might be prevented and patient safety improved.

### Data collection

The QMCI and the CQII were designed as data collection tools for use during on-site visits by experienced surveyors who had previous experience in hospital accreditation, but had no relationship with the hospitals. Data were collected in the 74 hospitals between May 2011 and February 2012. In total, 14 external surveyors (2 in each country) collected data. The surveyors were trained on the main aspects to be assessed, and the scoring system. A data collection manual was developed to provide guidance and ensure homogeneity of data collection. Data were first gathered on paper and then entered into an online database system and checked by the country coordinator for missing data. Every hospital was visited by two surveyors for 1 day. No hospital professionals were made aware of the contents of the visit beforehand.

### Data analysis

We began the analysis by describing the sample of hospitals that provided data from external visits. Then, we investigated the factor structure, reliability and construct validity of QMCI and CQII using standard psychometric methods. We conducted principal components, confirmatory factor, reliability coefficient, item-scale total correlation and inter-scale correlation analyses separately for QMCI and CQII. There were no missing values for any of the items, as data collected from hospitals participating in external visits were complete. Since we had external visit data from only 74 hospitals and factor analysis required 5–10 observations per variable, we did not split the data into two parts to perform factor analysis. We explored factor structure using principal component analysis with oblique (promax) rotation with a factor extraction criterion of eigenvalues >1 and three or more item loadings. Items were assigned to the factor where they had the highest factor loading, and only items with loadings ≥0.3 were retained. However, only one item was used to assess the ‘quality planning’ domain for QMCI, and this was considered to be theoretically important for assessing quality compliance. We then used confirmatory factor analysis to examine whether the data supported the final factor structure, where root mean square residual <0.05 and a non-normed fit index >0.9 indicated good fit. We also used Cronbach's alpha to assess internal consistency reliability of each factor, where a value of 0.7 was acceptable. Item-total correlations corrected for item overlap were used to examine the homogeneity of each scale. Item-total correlation coefficients of 0.4 or greater suggested adequate scale homogeneity. Lastly, we assessed the degree of redundancy between scales using inter-scale correlation coefficients, where a Pearson's correlation coefficient <0.7 was indicative of non-redundancy. Once psychometric evaluations of QMCI and CQII were completed and a final factor structure was established, we computed scores for each of the scales comprising these indices by taking the mean of items retained for each scale from the factor analysis. These sub-scales were then summed to build each final index. We subtracted the number of scales from the final CQII in order to bring the lower bound of the index down to 0. In order to assess construct validity, we used Pearson's correlation coefficients to examine the relationship between CQII and QMCI. We also provide descriptive statistics on the final index, sub-scales aggregated to build the index and items that comprise the sub-scales. All statistical analyses were carried out in SAS (version 9.3, SAS Institute, Inc., NC, 2012).

## Results

### Hospital characteristics

Across the 7 countries, 74 hospitals participated in this in-depth part of the DUQuE project. The teaching status was evenly balanced between non-teaching (55%) and teaching (45%). Most hospitals were publicly owned (80%) and comprised 501–1000 beds (42%) (Table 1).

**Table 1:** Characteristics of hospitals participating in the analysis

Characteristic	*n*	%
Number of hospitals	74	100
Teaching status, *n* (%)		
* *Teaching	33	45
* *Non-teaching	41	55
Ownership, *n* (%)		
* *Public hospitals	59	80
* *Private (or mixed ownership)	15	20
Approximate number of beds in hospital		
* *<200	7	9
* *200–500	22	30
* *501–1000	31	42
* *>1000	14	19

### Quality Management Compliance Index

The QMCI was designed to measure compliance in 3 domains: quality planning (1 item); quality control and monitoring (12 items) and improving quality by Staff development (5 items), but factor analysis revealed 4 factors instead of the proposed three, as can be seen in Table 2. The quality planning factor comprised one item as this domain was only assessed with one question in our questionnaire. The other three QMCI sub-scales were monitoring of patient/professional opinions, monitoring of quality systems and improving quality by staff development. Factor analysis revealed that the items initially included in the quality control and monitoring domain actually clustered on two distinct factors: the monitoring of the opinions of patients and professional and that of quality systems. This distinction is meaningful and was retained. Three items from the questionnaire did not load on any of the factors and these were excluded.

**Table 2:** Factor loadings, Cronbach's alpha and corrected item-total correlations of QMCI (*n* = 74)

Scale and items of QMCI	Factor loadings on primary scale	Internal consistency reliability: Cronbach's *α*	Corrected item-total correlation
Quality planning		–	
Q1 The hospital (management) board approved an annual programme for quality improvement in 2010	–		–
Monitoring of patient/professional opinions		0.742	
Q2 The results of patient satisfaction surveys were formally reported to the hospital (management) board in 2010	0.534		0.450
Q3 The hospital (management) board received results of surveys of staff satisfaction in 2010	0.522		0.411
Q4 Patients incidents and adverse events are analysed and evaluated	0.585		0.491
Q5 Patients' opinion/perception is measured and evaluated	0.553		0.474
Q6 Patient complaint system is available and/or evaluated	0.534		0.440
Q7 Professional opinion/perception is measured and evaluated	0.692		0.606
Monitoring of quality systems		0.783	
Q8 The hospital (management) board received regular, formal reports on quality and safety in 2010	0.720		0.593
Q9 Medical leaders received regular, formal reports on quality and safety in 2010	0.763		0.651
Q10 There is an active clinical guidelines register	0.671		0.607
Q11 Guidelines application are measured and evaluated	0.577		0.505
Improving quality by staff development		0.756	
Q12 The hospital maintains a record for each member of the medical staff that contains a copy of documents related to license, education, experience and certification	0.876		0.674
Q13 The hospital maintains a record for each member of the nursing staff that contains a copy of documents related to license, education, experience and certification	0.847		0.623
Q14 The performance of all individual medical staff members is formally reviewed to determine continued competence to provide patient care services	0.553		0.545
Q15 The performance of all nursing staff members is formally reviewed to determine continued competence to provide patient care services	0.402		0.387

The factors of the QMCI yielded acceptable results with regard to internal consistency (Cronbach's alpha ranges between 0.74 and 0.78). None of the corrected item-total correlations were <0.4, except in the case of one item in the improving quality by staff development sub-scale, indicating that all items contribute to the distinction between high and low scores on the factor. The inter-scale correlations, presented in Table 3, had a maximum of 0.52, which is below the maximum threshold of 0.70. This indicates that the QMCI is indeed a multi-dimensional construct with sub-scales addressing independent aspects of quality management. All sub-scales had notable correlations with the overall index, meaning that they contribute to the QMCI.

**Table 3:** Inter-scale correlation coefficients of QMCI and scales with overall construct (*n* = 74)

	1.	2.	3.	4.	QMCI
1. Quality planning	1				0.78
2. Quality control and monitoring of patient/professional opinions	0.317	1			0.69
3. Quality control and monitoring of quality systems	0.520	0.475	1		0.78
4. Improving quality by staff development	0.142	0.303	0.145	1	0.52

Note. The numbers in the first row correspond to the scales in the first column.

Descriptive statistics for QMCI, the sub-scales and items that comprise the sub-scales are presented in Table 4. QMCI had a final scale range of 0–16. Six out of 15 items had a median score of 4 (range 0–4). This is also reflected in a high ceiling ratio of these items. In contrast to most other items, the third item (improving quality by staff development) was an exception. It had a low average (zero) and a high floor ratio.

**Table 4:** Distribution of item and scale scores of QMCI (*n* = 74)

Scale and items of QMCI (range 0–4)	Median (IQR)^a^	Floor (% with lowest score)	Ceiling (% with highest score)
Quality Management Compliance Index (QMCI)^b^ (range 0–16)	10 (3.2)		
Quality planning; mean (SD)	2.9 (1.4)		
Q1 The hospital (management) board approved an annual programme for quality improvement in 2010	4 (2)	14	58
Monitoring of patient/professional opinions; mean (SD)	2.7 (0.8)		
Q2 The results of patient satisfaction surveys were formally reported to the hospital (management) board in 2010	4 (2)	7	57
Q3 The hospital (management) board received results of surveys of staff satisfaction in 2010	2 (4)	34	28
Q4 Patients incidents and adverse events are analyzed and evaluated	3 (3)	19	34
Q5 Patients' opinion/perception is measured and evaluated	4 (1)	1	65
Q6 Patient complaint system is available and/or evaluated	4 (1)	1	69
Q7 Professional opinion/perception is measured and evaluated	2 (4)	35	34
Monitoring of quality systems; mean (SD)	2.1 (1.1)		
Q8 The hospital (management) board received regular, formal reports on quality and safety in 2010	3 (2)	14	39
Q9 Medical leaders received regular, formal reports on quality and safety in 2010	3 (2)	15	39
Q10 There is an active clinical guidelines register	2 (4)	27	27
Q11 Guidelines application are measured and evaluated	1 (2)	32	18
Improving quality by staff development; mean (SD)	2.4 (1.0)		
Q12 The hospital maintains a record for each member of the medical staff that contains a copy of documents related to license, education, experience and certification	4 (2)	3	61
Q13 The hospital maintains a record for each member of the nursing staff that contains a copy of documents related to license, education, experience and certification	4 (2)	3	61
Q14 The performance of all individual medical staff members is formally reviewed to determine continued competence to provide patient care services	0 (2)	61	18
Q15 The performance of all nursing staff members is formally reviewed to determine continued competence to provide patient care services	2 (4)	34	38

^a^Median (IQR) presented for individual question items.

^b^QMCI is the sum of all 4 sub-scales, range: 0–16.

### Clinical quality implementation index

The CQII aimed to assess three levels of implementation, such as existence of protocol, monitoring of compliance and sustainability by measuring and using indicators to keep an improvement focus. Factor analysis revealed, however, that the items did not group into these dimensions. Instead, the factors appear to be grouped according to different clinical areas (Table 5) suggesting that the levels of clinical implementation are not consistent on the same level across different clinical areas. Rather, the levels of development coexist and reflect the implementation of a certain area. Therefore, we used the items to describe clinical implementation as a single score for each area. The seven sub-scales retained by factor analysis were preventing hospital infection, medication management, preventing patient falls, preventing patient ulcers, routine testing of elective surgery patients, safe surgery practices and preventing deterioration. The resulting seven-factor structure showed high factor loadings, Cronbach's alphas ranging from 0.82 to 0.93 and corrected item-total correlations. The inter-scale correlations, presented in Table 6, had a maximum of 0.59, which is below the maximum threshold of 0.70. This indicates that the CQII is a multi-dimensional construct.

**Table 5:** Factor loadings, Cronbach's alpha and corrected item-total correlations of CQII (*n* = 74)

Scale and items of CQII	Factor loadings on primary scale	Internal consistency reliability: Cronbach's *α*	Corrected item-total correlation
Preventing hospital infection		0.817	
C1 Responsible group exists	0.574		0.522
C2 Hospital protocol exists	0.548		0.491
C3 Extent of compliance monitoring	0.833		0.789
C4 Sustainability of the system	0.808		0.693
C5 Improvement focus	0.719		0.558
Medication management		0.903	
C6 Responsible group exists	0.671		0.655
C7 Hospital protocol exists	0.567		0.554
C8 Extent of compliance monitoring	0.954		0.899
C9 Sustainability of the system	0.938		0.855
C10 Improvement focus	0.917		0.845
Preventing patient falls		0.898	
C11 Responsible group exists	0.610		0.590
C12 Hospital protocol exists	0.681		0.648
C13 Extent of compliance monitoring	0.907		0.859
C14 Sustainability of the system	0.952		0.890
C15 Improvement focus	0.850		0.772
Preventing patient ulcers		0.879	
C16 Responsible group exists	0.644		0.612
C17 Hospital protocol exists	0.631		0.600
C18 Extent of compliance monitoring	0.867		0.810
C19 Sustainability of the system	0.889		0.807
C20 Improvement focus	0.804		0.733
Routine testing of elective surgery patients		0.923	
C21 Responsible group exists	0.581		0.571
C22 Hospital protocol exists	0.702		0.679
C23 Extent of compliance monitoring	0.984		0.937
C24 Sustainability of the system	0.983		0.929
C25 Improvement focus	0.970		0.910
Safe surgery practices		0.881	
C26 Responsible group exists	0.647		0.616
C27 Hospital protocol exists	0.537		0.513
C28 Extent of compliance monitoring	0.918		0.850
C29 Sustainability of the system	0.887		0.812
C30 Improvement focus	0.867		0.809
Preventing deterioration		0.932	
C31 Responsible group exists	0.804		0.774
C32 Hospital protocol exists	0.787		0.757
C33 Extent of compliance monitoring	0.905		0.868
C34 Sustainability of the system	0.891		0.850
C35 Improvement focus	0.895		0.855

**Table 6:** Inter-scale correlation coefficients (*n* = 74)

	1	2	3	4	5	6	7
1. Preventing hospital infection	1.000						
2. Medication management	0.585	1.000					
3. Preventing patient falls	0.130	−0.019	1.000				
4. Preventing patient ulcers	0.249	0.285	0.391	1.000			
5. Routine testing of elective surgery patients	0.170	0.204	0.168	0.371	1.000		
6. Safe surgery practices	0.364	0.426	0.227	0.182	0.276	1.000	
7. Preventing deterioration	0.371	0.258	0.285	0.053	0.160	0.452	1
Overall construct CQII	0.59	0.60	0.55	0.60	0.57	0.70	0.63

CQII had a final scale range of 0–14. The distribution of the scores (Table 7) showed that the prevention of hospital infection stands out with a very high average score and a ceiling ratio of over 80% for all of its items. For other items it seems that quite a number of hospitals have the highest or lowest score (ceiling effects). Around two-thirds of the hospitals have a low score on the sub-scale routine testing of elective surgery patients.

**Table 7:** Distribution of item, scale and index scores (*n* = 74)

Scale and items (range 1–3)	Median (IQR)^a^	Floor (% with lowest score)	Ceiling (% with highest score)
Clinical Quality Implementation Index (CQII)^b^ (range 0–14)	8.3 (2.9)		
Preventing hospital infection; mean (SD)	2.8 (0.3)		
C1 Responsible group exists	3 (0.0)	5	88
C2 Hospital protocol exists	3 (0.0)	1	80
C3 Extent of compliance monitoring	3 (0.0)	1	93
C4 Sustainability of the system	3 (0.0)	3	89
C5 Improvement focus	3 (0.0)	5	86
Medication management; mean (SD)	2.4 (0.6)		
C6 Responsible group exists	3 (1.0)	16	72
C7 Hospital protocol exists	3 (1.0)	14	61
C8 Extent of compliance monitoring	3 (1.0)	20	61
C9 Sustainability of the system	3 (1.0)	20	64
C10 Improvement focus	3 (1.0)	23	55
Preventing patient falls; mean (SD)	2.1 (0.7)		
C11 Responsible group exists	2 (2.0)	50	39
C12 Hospital protocol exists	3 (1.0)	23	57
C13 Extent of compliance monitoring	3 (2.0)	30	55
C14 Sustainability of the system	2 (2.0)	38	49
C15 Improvement focus	2 (2.0)	46	45
Preventing patient ulcers; mean (SD)	2.3 (0.7)		
C16 Responsible group exists	3 (2.0)	30	59
C17 Hospital protocol exists	3 (1.0)	16	58
C18 Extent of compliance monitoring	3 (2.0)	26	62
C19 Sustainability of the system	3 (2.0)	28	59
C20 Improvement focus	3 (2.0)	35	57
Routine testing of elective surgery patients; mean (SD)	1.5 (0.7)		
C21 Responsible group exists	1 (1.0)	66	24
C22 Hospital protocol exists	1 (1.0)	59	22
C23 Extent of compliance monitoring	1 (1.0)	72	20
C24 Sustainability of the system	1 (1.0)	72	19
C25 Improvement focus	1 (1.0)	74	20
Safe surgery practices; mean (SD)	2.1 (0.7)		
C26 Responsible group exists	3 (2.0)	43	50
C27 Hospital protocol exists	3 (1.0)	20	59
C28 Extent of compliance monitoring	2 (2.0)	42	46
C29 Sustainability of the system	2 (2.0)	45	45
C30 Improvement focus	1 (2.0)	51	35
Preventing deterioration; mean (SD)	2.0 (0.8)		
C31 Responsible group exists	3 (2.0)	36	55
C32 Hospital protocol exists	2 (2.0)	32	45
C33 Extent of compliance monitoring	2 (2.0)	46	43
C34 Sustainability of the system	2 (2.0)	49	43
C35 Improvement focus	2 (2.0)	50	36

^a^Median (IQR) presented for individual question items

^b^CQII is the sum of all 7 sub-scales (minus 7), range: 0–14.

### Construct validity: hypothesis testing

The inter-index correlation between QMCI and the CQII was 0.565. This was in line with our expectations that both are distinct, but related constructs.

## Discussion

### Main findings

The results suggest that at the hospital level the QMCI and CQII are reliable and valid to assess compliance with quality management procedures as well as the extent of several activities related to continuous improvement of clinical quality. The latter activities included having a group of professionals responsible for the clinical area as well as a formally approved protocol and performance indicators. The initially proposed factor structure of both indices had to be adjusted based on the results of the factor analysis, but these minor adjustments did not change the theoretical constructs, instead refining the fit of the sub-scales to the concepts of interest. Both QMCI and CQII showed a high internal consistency and appeared to be multi-dimensional constructs. The descriptive results showed that some items included in the indices may be subject to ceiling effects with a large proportion of respondents having a positive score. Despite that, we kept the items and clinical areas in the instrument in case subsequent testing in across a broader range of hospitals in other European countries reveals more variation than those investigated in our study.

### Strength and limitations

On-site audits have the advantage that they provide more objective and independent outcomes based on factual information derived for instance from an annual report. In contrast to self-administered questionnaires, audit can avoid potential social desirability bias in responses. The time burden for the audited organization is relatively low compared with an all staff survey. The downside is that documents on which the audit is based have to be reliable. Furthermore, trained surveyors are needed to conduct the audit to minimize variation due to inter-observer differences.

### Relation with other studies

Using audit as a measurement strategy is relatively rare, although it may grow as it is proving useful in other study designs [4, 5]. More often audit instruments are used as a tool to improve quality, especially in the form of an accreditation programme. As a recent review suggests, audits seem to lead to improved structure and process of care and even clinical outcomes [6].

## Conclusion

The two indices we developed and evaluated have the potential for use in research and in routine practice to help hospitals focus on quality and safety issues as well as follow the quality improvement P-D-C/S-A cycle. The instruments can also be used by purchasers, policy-makers or health-care inspectorates, if they want to assess the implementation of quality management at hospitals level in a more standardized way. The QMCI focuses on the core elements of a quality system, while the CQII has its focus on clinical areas that are directly related to patient care at ward level. Future research is needed to investigate the relationship between these novel quality measurement tools and other indicators including patient outcomes.

## Funding

Deepening our Understanding of Quality Improvement in Europe (DUQuE) has received funding from the European Community's Seventh Framework Programme (FP7/2007–2013) under grant agreement number 241822. Funding to pay the Open Access publication charges for this article was provided by European Community's Seventh Framework Programme (FP7/2007–2013) under grant agreement no. 241822.
